# Supplementary material for: SUMOylated SNF2PH promotes variant surface glycoprotein expression in bloodstream trypanosomes
Source: EMBO Rep. 2019 Nov 6;20(12):e48029. doi: 10.15252/embr.201948029 (PMC6893287; doi:10.15252/embr.201948029)
Supplement: Supplementary file 1 — Appendix [file EMBR-20-e48029-s001.pdf]

Appendix for:

A homeodomain protein SNF2PH promotes VSG and bloodstream stage gene expression in African trypanosomes

Andreu Saura, Paula Iribarren, Domingo Rojas-Barros, Jean M. Bart, Diana López-Farfán, Eduardo Andrés-león, Isabel Vidal-Cobo, Cordula Boehm, Vanina Alvarez, Mark C. Field and Miguel Navarro.

Table of contents

Supplementary Tables 2

Appendix Table S1. SUMO-target proteins 2

Appendix Table S2. Proteins associated with SNF2PH. 3

Appendix Table S3. Genes expressed after SNF2PH depletion. 4

Appendix Table S4. Primers used for RT-qPCR and ChIP-qPCR. 5

Appendix Table S5. Primers used for cloning and diagnosis. 6

Appendix Table S6. Plasmids used in this work. 7

Supplementary Figures 8

Appendix Figure S1. The SNF2 N and helicase domains are conserved in trypanosome SNF2PH whilst PH domain is normally included in methyltransferases. 8

Appendix Figure S2. SNF2PH is differentially expressed in both developmental stages. 9

Appendix Figure S3. *In situ* detection of SUMOylated SNF2PH using a Proximity Ligation Assay (PLA) 10

Appendix Figure S4. SNF2PH depletion effect at inactive telomeric VSGs is clone-dependent. 11

Appendix Figure S5. Downregulation of SNF2PH during stumpy form development. 12

Appendix Tables

Appendix Table S1. SUMO-target proteins.

Accession Number	Description
Tb927.3.2140	Transcription Activator
Tb927.10.1610	Hypothetical protein conserved
Tb927.11.4380	ATP-dependent RNA helicase
Tb927.10.13900	UDP-galactose transporter
Tb927.6.4430	Homoserine kinase (HK)
Tb927.6.3150	Hydin flagellar component
Tb927.8.2450	5-AMP-activated protein kinase beta subunit
Tb927.11.13930	U3 small nucleolar ribonucleoprotein protein IMP
Tb927.11.14190	Tudor domain containing protein
Tb927.8.5640	Succinate dehydrogenase subunit
Tb927.10.13280	Hypothetical protein with HEAT repeat
Tb927.5.1560	ATP-dependent DEAD/H RNA helicase
Tb927.5.2420	NOL1/NOP2/sun family (Methyltransferase activity)
Tb927.11.1420	Hypothetical protein with WD40 repeat (Interaction E3 Ligases)
Tb927.6.4770	Protein mkt1
Tb927.10.7510	Chromatin binding protein
Tb927.6.4750	Hypothetical protein with WD40 repeat (Microtubule-based movement)
Tb927.4.410	CAF 40
Tb927.8.8140	Small GTP-binding rab protein
Tb927.11.9890	Signal recognition particle receptor alpha subunit
Tb927.8.4500	Eukaryotic translation initiation factor 4 gamma 5
Tb927.2.4950	Hypothetical protein
Tb927.9.12490	Hypothetical protein with WD40 repeat (Calcium-binding EF-hand)
Tb927.2.4230	NUP-1 protein
Tb927.9.11500	Anaphase-promoting Complex Subunit 2
Tb927.11.4380	ATP-dependent RNA helicase
Tb927.10.7490	Cullin 2
Tb927.10.8420	Target of rapamycin (TOR) kinase 1
Tb927.11.15830	RNA methyltransferase
Tb927.4.470	snoRNP protein GAR1
Tb927.11.10190	Telomerase reverse transcriptase
Tb927.10.11020	DNA mismatch repair protein MSH2

SUMO-target proteins identified in two independent proteomic analyses by tandem affinity purification (histidine/HA).

Appendix Table S2. Proteins associated with SNF2PH.

Accession Number	Description	Number of peptides
Tb927.3.2140	Transcription activator, putative	68
Tb927.1.1380	Serine/threonine protein phosphatase 2A regulatory subunit, putative	14
Tb927.3.1300	Hypothetical protein, conserved (posttranscriptional regulation of gene expression)	12
Tb927.7.6810	EF-hand domain pair, putative	9
Tb927.3.5620	Facilitates chromatin transcription complex (FACT) subunit spt16	8
Tb927.7.2680	Zinc finger protein family member, putative (ZC3H22)	7
Tb927.10.4070	Hypothetical protein, conserved	6
Tb927.2.5240	pre-mRNA splicing factor 19 (TbPRP19)	4
Tb927.10.4440	Predicted SAP domain protein	4
Tb927.11.4910	Predicted ankyrin repeat family protein	4
Tb927.11.12790	Ribonucleoside-diphosphate reductase small chain (RNR2)	4
Tb927.11.5650	Replication factor C, subunit 1, putative,replication factor C	4
Tb927.3.2440	AGC essential kinase 1 (AEK1)	4
Tb927.8.1990	Peroxidoxin	4
Tb927.7.4810	HD domain containing protein, putative	3
Tb927.3.1240	Serine/threonine-protein phosphatase 2A, putative	3
Tb927.9.9230	Hypothetical protein, conserved	3
Tb927.3.1900	Conserved protein, unknown function (Midasin AAA ATPase)	2
Tb927.10.540	TP-dependent RNA helicase SUB2, putative	2
Tb927.11.13090	Elongation factor 1 gamma, putative	2
Tb927.11.4900	WD40/YVTN repeat-like-containing protein	2
Tb927.9.5190	Proliferative cell nuclear antigen (PCNA), putative	2
Tb927.10.15180	Nucleosome assembly protein	2
Tb927.11.630	RNA polymerase I second largest subunit (RPA135)	2
Tb927.4.5020	RNA polymerase IIA largest subunit (RPB1)	2
Tb927.11.8310	Class I transcription factor A, subunit 4 (CITFA-4)	2

Proteins associated with SNF2PH in procyclic forms. Partners were selected by a high number of peptides identified by LC-MS/MS. Contaminant proteins from a negative non-tagged sample were subtracted from the list.

Appendix Table S3. Genes expressed after SNF2PH depletion.

GeneName	Length	logFC	logCPM	F	FDR	Description
Tb427.03.2140	2847	-1.73	6.18	221.44	1.54E-45	Transcription activator
Tb427.07.2820	405	0.55	7.78	33.25	3.64E-05	histone H2A
Tb427.06.510	345	1.08	4.77	25.39	1.38E-03	GPEET2 procyclin precursor
Tb427_09_v4.sno RNA.0026	96	1.52	1.77	22.69	4.20E-03	C/D snoRNA
Tb427.01.4710	537	1.16	4.24	20.80	8.90E-03	hypothetical protein
Tb427.BES40.15	2031	0.42	8.50	20.46	8.90E-03	expression site-associated gene 8 (ESAG8) protein
Tb427.10.10910	2115	-0.32	11.87	19.64	1.17E-02	heat shock protein, putative
Tb427.10.10240	1218	1.28	1.66	19.05	1.40E-02	procyclin-associated gene 1 (PAG1) protein
Tb427.10.10260	426	0.95	4.52	18.01	1.94E-02	EP1 procyclin
Tb427.BES40.18	1413	0.37	9.94	17.97	1.94E-02	expression site-associated gene 2 (ESAG2) protein
Tb427.BES40.14	1893	0.39	8.73	17.81	1.94E-02	expression site-associated gene 8 (ESAG8) protein
Tb427.10.10230	1113	1.22	1.73	17.28	2.35E-02	procyclin-associated gene 5 (PAG5) protein
Tb427.10.7160	621	1.20	0.06	17.05	2.46E-02	procyclin-associated gene 1 (PAG1) protein
Tb427.BES64.2	1467	1.13	3.50	16.70	2.67E-02	variant surface glycoprotein (VSG)
Tb427.10.10210	1122	1.17	1.30	16.62	2.67E-02	procyclin-associated gene 4 (PAG4) protein
Tb427.10.10250	390	1.02	4.10	16.33	2.91E-02	EP2 procyclin
Tb427.10.10960	2115	-0.28	12.19	16.18	2.97E-02	heat shock protein, putative
Tb427.10.10970	2115	-0.27	12.56	15.80	3.42E-02	heat shock protein, putative

Genes expressed after SNF2PH depletion. Displayed genes were identified from two biological replicates with a FDR<0.05 and P-value <7.09E^{-05}. Representative values are expressed as a log fold change (logFC), log for count per million (logCPM) and sorted by the highest F score.

Appendix Table S4. Primers used for RT-qPCR and ChIP-qPCR.

Gene or Region	Primer name	Sequence (5´-3`)
1	ESMP1_U	GGTGTGGCGGACGTCTCGAAC
	ESMP1_L	CCTCTAAAATACGCTCAGCCCGTCC
4	ESMP4_U	CGGAGAATATTTCGGATGC
	ESMP4_L	AATCGTTACGGCCAAATTCA
VSG221 (MITat1.2/BES 1)	VSG221_U	AGCTAGACGACCAACCGAAGG
	VSG221_L	CGCTGGTGCCGCTCTCCTTTG
VSG121 (MITat1.2/BES 3)	VSG121_U	CCTGACATCGGACGGTAAC
	VSG121short_L	TGGTCGTATTTGCCTTCCTT
VSGJS1 (MITat1.2/BES 13)	VSGJS1_U	TTCTGCTTCTTTGCCCTTGT
	VSGJS1_L	AAAATGAAGCGGAAATGGTG
VSGVO2 (MITat 1.2/BES 2)	VSGVO2 short_U	ACAGAATCGGCCACAGAAAG
	VSGVO1_L	CATTTCCGCGTTGTCTTGTA
BC118 (Tb927.4.4460)	BC118_U	CAGAAGCGCCAATACAACAA
	BC118_L	CGTTAGAAACCACGCCAGTT
BC222 (Tb927.8.7070)	BC222_U	CGGAAGAGACAATTCGAAGG
	BC222_L	CGCCATTGACATCCCTACTT
18S (Tb927_01.rRNA.1)	18S_U	GACGTAATCTGCCGCCAAAAT
	18S_L	AACGCCATGGCAGTCCAGTAC
rDNA pro	rDNAprom_U	GTCAATACAACACACAATAGG
	rDNApro_L	CTTAACTGAGGAAGTGTCATA
rDNA sp (927/4 GUTat10.1)	rDNAspacer_U	ATTTTCTCTACCCCTCTCTT
	rDNAspacer_L	ATCATCGTATCATTTTCATC
EP cds (Tb927.6.520)	EP3-2U	ATGGCACCTCGTTCCCTTTA
	EP3-2L	AGAATGCGGCAACGAGACCAA
EP pro	ProcyProm_U	AGTTTAAGATGTTCTCGTGAT
	ProcyProm_L	CTTTTTGGTGTAATTGAAGTC
FLuc	Luc_U	GTGTTGGGCGCGTTATTTAT
	Luc_L	CATCGACTGAAATCCCTGGT
RLuc	Ren_U	GATAACTGGTCCGCAGTGGT
	Ren_L	ACCAGATTTGCCTGATTTGC
SL (Tb927_09_v4)	SL_U	CCGACACGTTTCTGGCACGACAG
	SL_L	TGCGTGTGTTGGCCCAGCTGCTAC
SL Pro	SL Pro_U	ATAATGCATGACCCCTTTGTTTCCCATAA
	SLPro_L	ATAGATCTCAGAAACTGTTCTAATAATAGCGTTAGTTG
Tubulin (Tb927.1.2330)	Tub_U	AGGCAACGGGAGGTCGCTATG
	Tub_L	GGGATGGGATGATGGAGAAAG
Myosin B (Tb927.11.16310)	MyoB_U	CTGCAGAACAAGCACGGCATT
	MyoB_L	ACGCTCAACAGTGGCAGTGAA
U2 (Tb927.10.14360)	U2_U	CTGCGTGATCTTTTGTTCCT
	U2_L	CGACTGCGTTTTCGTATTTT
C1 (Tb10.389.0540)	C1_U	TTGTGACGACGAGAGCAAAC
	C1_L	GAAGTGGTTGAACGCCAAAT
5S (Tb927.8.1384)	5Snew_U	GACCATACTTGGCCGAATG
	5Snew_L	TACAACACCCCGGGTTCC
rDNA+780 (927/4 GUTat10.1)	rDNA_705 U	ATTGCCGCTGCTTTTTACAC
	rDNA_890 L	TATCAGGTGCCAAGCCCTAC
PAD1 (Tb927.7.5930)	PAD1_U	TCATGGTTTCGCCATTCTCGTAACC
	PAD1_L	CTCAGCCACTTCTCTCCTACAACAC
PAD2 (Tb927.7.5940)	PAD2_U	AGGGTGATGCCAAAGAACAC
	PAD2_L	TACCCACACCGTTGAGAACA
AMPKa1 (Tb927.10.5310)	C1_U	GGATCCAGGCAAATGACCAAG
	C1_L	GTCCCTCTTCTACCTTTACAG
SNF2PH (Tb927.3.2140)	Snf2_qPcr_U	AGTCCCGTGAAGGTCCTCTT
	Snf2_qPcr_L	CTTGTCGAACTGGTGGGTCT
prom SSR7_3U	3U_F	AATACAGCGGTGACTGTCA
	3U_R	AAAAAAAAAGCGAGCAATAATGA

Appendix Table S5. Primers used for cloning and diagnosis. Sequences from each oligonucleotide pair are shown with their respective enzyme restriction site at 5' end (underlined). N-T: Amino-terminal. C-T: Carboxyl-terminal. HA: Hemagglutinin.

Oligonucleotide name	Sequence (5'-3')	Gene	Restriction enzyme end	Application
SNF2PH-c2PrC_U	CGGGATCCGAGCTCTCGATGGCGCGTT TCAACGG	C-T SNF2PH	*Bam*HI	RNAi / His tag
SNF2PH-c2PrC_L	CAAGCTTGGTTAACGTGAGATTCACTGA AACTGCC	C-T SNF2PH	*Hind*III	RNAi / His tag
nSnf-2_NcoI_U	TCCCCATGGCTATGAAGTTTGAGAACCC CGTT	N-T SNF2PH	*Nco*I	Flag tag
nSnf-2_NcoI_L	AACCCATGGAAATTGGACTCCATCGAAC TGGTA	N-T SNF2PH	*Nco*I	Flag tag
cSnf-2_NcoI_ atg_U	TCCCCATGGCTATGCACCGCGACGGAC TAACTGAA	C-T SNF2PH	*Nco*I	Flag tag
cSnf-2_NcoI_L	AACCCATGGGTGAGATTCACTGAAACTG CC	C-T SNF2PH	*Nco*I	Flag tag
SNF2-haNter-U	GCCGCTCAGCTGGGATCCGGCATGAAG TTTGAGAACCCCGTT	N-T SNF2PH	*Blp*I	HA tag
SNF2-haNter-L	GCAAGCTTCTCGAGCCGCCGTGCTTTCC TTCAGCAT	N-T SNF2PH	*Xho*I	HA tag
SNF2-PHD-U	AACGCTCAGCTGGGCATGAACAGGGAA GAACACTGGAGT	PHD	*Blp*I	HA tag
3'UTR-SNF2-PHD_U	GTTATCGCCACGTCATTCGTACTTA	3'UTR PHD	—	Diagnosis
3'SNF2_ΔPHD-L	GCAGGATCCCTATCTAGTAAGAGAGTCC CGCCC	3'UTR ΔPHD	*Bam*HI	HA tag
5SNF2-New-U	GTTAACGCTCAGCTGGGCATGAAGTTTG AGAACCCCGTT	Full length SNF2PH	*Blp*I	HA tag
3SNF2_Bam-L	AGGATCCCTAGTGAGATTCACTGAAACT	Full length SNF2PH	*Bam*HI	HA /3 x HA tags
Hyg_L	CAATAGGTCAGGCTCTCGCTGAAT	Hygromycin	—	Diagnosis
Bla_L	CAGAGATGGGGATGCTGTTGATTG	Blasticidin	—	Diagnosis
3HA_U	ACAAGCTTGATGTACCCATACGATGTGC CGGATTACGCTGGGAGCTACCCATACGA TGTGCCGGATTACGCTGGCTACCCATAC GATGTGCCA	3 x HA	*Hind*III	3 x HA tag

Appendix Table S6. Plasmids used in this work. Plasmid constructs generated for *T. brucei* transfection and heterologous protein expression in *E. coli*.

Plasmid	Cloning strategy
p2T7Bla	Phleomycin marker from p2T7[i] was substituted by Blasticidin used to generate an RNAi from a bidirectional T7 promoter.
p2T7-cSNF2PH-Bla	The PCR product corresponding to 1113-bp of C-T SNF2PH using the oligonucleotide pair **SNF2PH-c2PrC_U /SNF2PH-c2PrC_L** was cloned into the *Bam*HI / *Hind*III restriction sites of p2T7Bla.
pET28a(+)- 6xHis tag	Expression plasmid in *E.coli* to produce an inducible 6 x Histidine fusion protein.
pET28a(+)- 6xHis-cSNF2PH	The PCR product corresponding to 1113-bp of C-T SNF2PH using the oligonucleotide pair **SNF2PH-c2PrC_U /SNF2PH-c2PrC_L** was cloned into the *Bam*HI / *Hind*III restriction sites of pET28a(+)- 6xHis to allow the heterologous expression in *E.coli.*
pET28a(+)-3xFlag	Expression plasmid in *E.coli* to produce an inducible 3 x Flag fusion protein.
pET28a(+)-3xFlag-SNF2PHN	The PCR product corresponding to 735-bp of N-T SNF2PH using the oligonucleotide pair **nSnf-2_NcoI_U /nSnf-2_NcoI_L** was cloned into the *Nco*I restriction sites of pET28a(+)-3xFlag to allow the heterologous expression of the substrate protein in *E. coli* for the *in vitro* SUMOylation system.
pET28a(+)-3xFlag-SNF2PHC	The PCR product corresponding to 705-bp of C-T SNF2PH using the oligonucleotide pair **cSnf-2_NcoI_atg_U/cSnf-2_NcoI_L** was cloned into the *Nco*I restriction sites of pET28a(+)-3xFlag.
pTAP-HA-H2Bv-Hyg	Expression plasmid in *T. brucei* targeted to the ribosomal spacer to ectopically express the H2Bv gene under an inducible T7 (procyclin) promoter.
pTAP1-RPB7cBla-Overex	Expression plasmid in *T. brucei* to express the RPB7 gene, used as a backbone for Blasticidine selection marker.
pTAP-HA-nSNF2PH-Hyg	The N-T fragment of SNF2PH (1068-bp) was amplified using the oligonucleotide pair **SNF2-haNter-U/SNF2-haNter-L** and cloned into the *Blp*I/*Xho*I sites of pTAP-HA-H2Bv-Hyg and expressed in *T. brucei* by homologous recombination (read through) under an inducible T7 promoter.
pTAP-HA-nSNF2PH-Bla	Expression plasmid containing the N-T fragment of SNF2PH (read through) with Blasticidine marker, obtained by cloning the HA SNF2 N-T gene (*Nsi*I/*Xho*I digested) derived from pTAP-HA-nSNF2PH-Hyg into the *Nsi*I/*Xho*I restriction sites of pTAP1-RPB7cBla-Overexp, used as backbone.
pTAP-HA-SNF2PH-FL-Hyg	Plasmid generated to ectopically express the full-length SNF2PH fragment under an inducible T7 promoter. The PCR product corresponding to the 2847-bp of the SNF2PH ORF was amplified using the oligonucleotide pair **5SNF2-New-U/3SNF2_Bam-L,** and cloned into the *Blp*I/*Bam*HI restriction sites of pTAP-HA-H2Bv-Hyg.
pTAP-HA-PHD-Hyg	Plasmid generated to ectopically express the PH domain of SNF2PH under an inducible T7 promoter. PCR product corresponding to the 747-bp fragment of PH domain was amplified using the oligonucleotide pair **SNF2-PHD-U/ 3SNF2_Bam-L** and cloned into the *Blp*I/*Bam*HI sites of pTAP-HA-H2Bv-Hyg.
pTAP-HA-SNF2ΔPHD-Hyg	Plasmid generated to ectopically express SNF2 helicase domain lacking PH domain (SNF2ΔPH) under an inducible T7 promoter. PCR product corresponding to the 1503-bp fragment of SNF2 domain was amplified using the oligonucleotide pair **5SNF2-New-U/ 3'SNF2_BamΔPHD-L** and cloned into the *Blp*I/*Bam*HI sites of pTAP-HA-H2Bv-Hyg.
p3HA-PHD-Hyg	Plasmid generated to ectopically express the 3xHA tagged PH domain of SNF2PH by cloning the 3xHA PHD gene fragment, amplified with the oligonucleotide pair **3HA_U/3SNF2_Bam-L** into the *Hind*III/*Bam*HI restriction sites of pTAP-HA-PHD-Hyg.
p3HA-PHD-Bla	Plasmid generated to ectopically express the 3xHA tagged PH domain with Blasticidine marker, by cloning the 3xHA PHD gene fragment (*Nsi*I/*Xho*I digested) derived from p3HA-PHD-Hyg into the *Nsi*I/*Xho*I restriction sites of pTAP-HA-nSNF2PH-Bla, used as backbone
p3HA-SNF2ΔPHD-Hyg	Plasmid generated to ectopically express the 3xHA tagged SNF2 helicase domain lacking the PH domain by cloning the SNF2ΔPHD gene fragment (*Blp*I/*Bam*HI digested) derived from pTAP-HA-SNF2ΔPHD-Hyg into the *Blp*I/*Bam*HI restriction sites of p3HA-PHD-Hyg.
p3HA-nSNF2PH-Hyg	Plasmid generated to express the 3xHA tagged N-T fragment of SNF2PH (read through) under an inducible T7 promoter, by cloning the HA nSNF2PH insert (*Blp*I/*Xho*I digested) derived from pTAP-HA-nSNF2PH-Hyg into the *Blp*I/*Xho*I restriction sites of p3HA-PHD-Hyg, used as backbone.
p3HA-nSNF2PH-Bla	Plasmid generated to express the 3xHA tagged N-T fragment of SNF2PH (read through) with Blasticidine marker, by cloning the 3xHA nSNF2PH insert (*Nsi*I/*Xho*I digested) derived from p3HA-nSNF2PH-Hyg into the *Nsi*I/*Xho*I restriction sites of p3HA-PHD-Bla, used as backbone.
p3HA-SNF2PH-FL-Hyg	Plasmid generated to ectopically express the 3 x HA tagged full length SNF2PH fragment under an inducible T7 promoter, by cloning the full length SNF2PH fragment (*Pvu*II/*Bam*HI digested) derived from pTAP-HA-SNF2PH-FL-Hyg into the *Pvu*II/*Bam*HI restriction sites of p3HA-PHD-Hyg, used as backbone.
pUC57- K2A-nSNF2PH	Synthetized plasmid (GeneScript) containing the N-terminal region of SNF2PH which includes a mutation of the lysine residue 2 into alanine (K2A), comprising *Pvu*II and *Sex*AI restriction sites in both 5'- and 3'- ends, respectively.
p3HA-K2A-nSNF2PH-Hyg	Plasmid generated to express the 3xHA tagged N-T SNF2PH K2A mutant (read through) by cloning the 412-bp of the N-terminal ORF with K2A mutation into the *Pvu*II/*Sex*AI restriction sites of p3HA-nSNF2PH-Hyg, used as backbone.
p3HA-K2A-nSNF2PH-Bla	Plasmid generated to express the 3xHA tagged N-T SNF2PH K2A mutant (read through) with Blasticidine marker, by cloning the N-T SNF2PH K2A insert (*Nsi*I/*Xho*I digested) derived from p3HA-K2A-nSNF2PH-Hyg into the *Nsi*I/*Xho*I restriction sites of p3HA-nSNF2PH-Bla, used as backbone.
pLEW100v5-8xHis-HA-TbSUMO	Plasmid generated to ectopically express the Histidine HA tagged version of TbSUMO under an inducible T7 promoter.

[i] *LaCount D.J., Bruse,S., Hill,K.L. and Donelson,J.E. (2000) Double-stranded RNA interference in Trypanosoma brucei using head-to-head promoters. Mol. Biochem. Parasitol., 111, 67–76.*

Appendix Figures

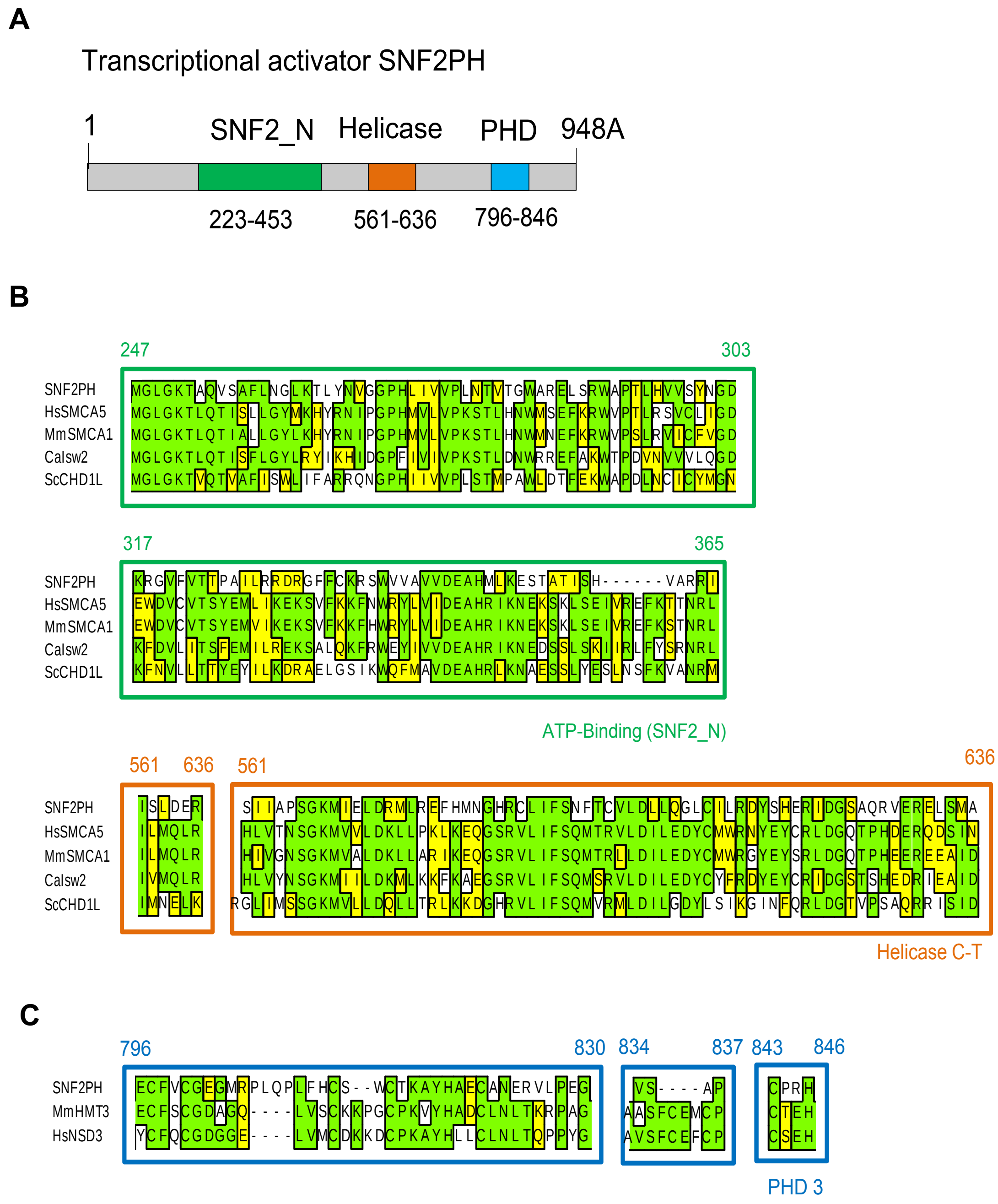

Appendix Figure S1. The SNF2 N and helicase domains are conserved in SNF2PH whilst PH domain is normally included in methyltransferases.

A. Schematic representation of the conserved regions of SNF2PH in other organisms. SNF2 N (green), Helicase C (orange) and PHD3 (blue).

B. Sequence alignment of SNF2 N (ATP-binding) and Helicase C-terminal domains of *T. brucei* SNF2PH (Tb927.3.2140), *H. sapiens* SMCA5 (O60264.1), *Mus musculus* SMCA1 (Q6PGB8.1), *M. musculus* CHD1L (Q9CXF7.1), *C. albicans* Isw2 (Q5A310.1) and *S. cerevisiae* CHD1L (P32657.1).

C. SNF2PH contains a PHD3 domain characteristic of HMTases. Sequence alignment of the PHD3 domain of *T. brucei* SNF2PH, *M. musculus* HMT3 (O88491) and *H. sapiens* NSD3 (Q9BZ95.1).

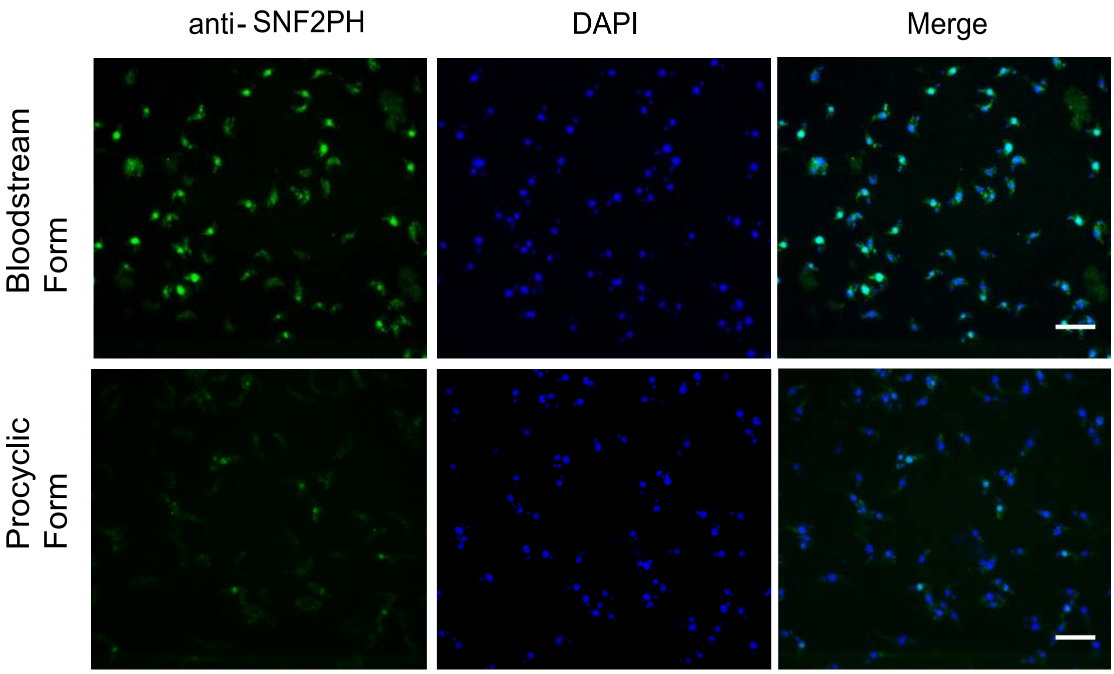

Appendix Figure S2. SNF2PH is differentially expressed in both developmental stages (p<0.001). Panels show DAPI stain and FITC chanel after IF using the anti-SNF2PH mAb and an anti-Mouse IgG Alexa Fluor 488 secondary Ab. Bars, 15 µm.

A

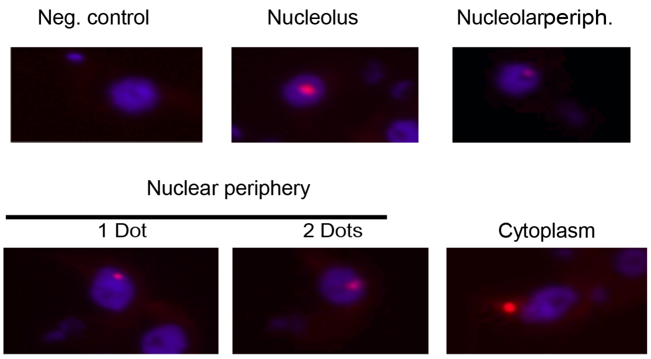

B

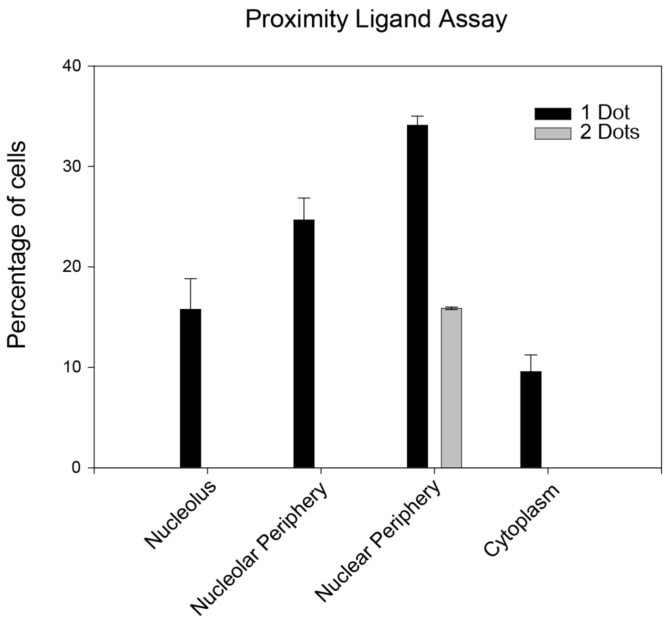

Appendix Figure S3. In situ detection of SUMOylated SNF2PH using Proximity Ligation Assay (PLA).

- **A.** SNF2PH SUMOylation was detected by in situ PLA (red dot) in bloodstream form cells using anti-SNF2PH rabbit antiserum and anti-TbSUMO mouse monoclonal antibody. SNF2PH appears to be SUMOylated in either nucleolus and nuclear periphery (1 or 2 Dots). Dots in cytoplasm are artefactual signals.

- **B.** Histogram showing the percentage of positive amplification signal detected in each compartment. Positive cells = 22.2±5.5% (n=2).

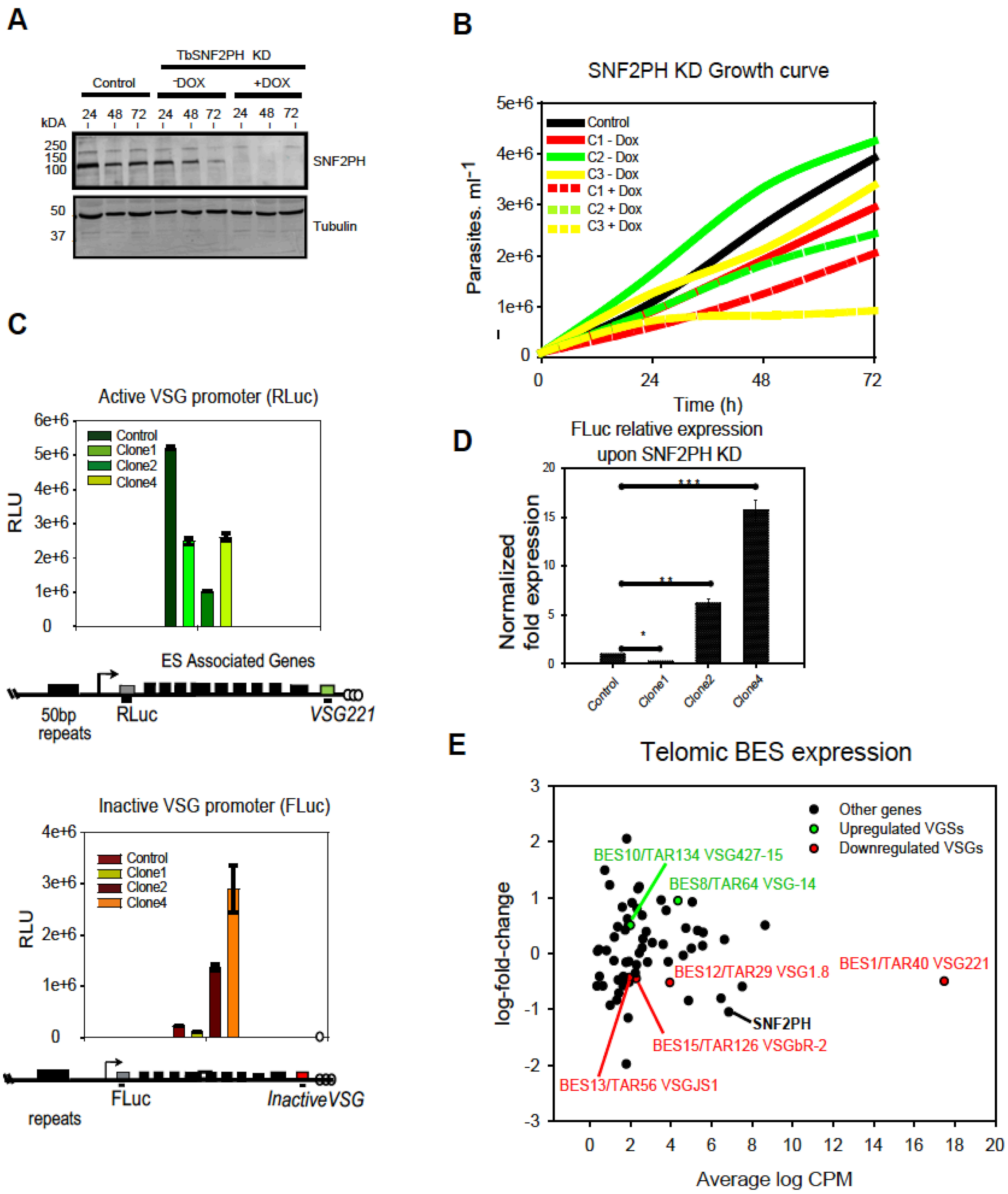

Appendix Figure S4. SNF2PH depletion effect at inactive telomeric VSGs is clone-dependent.

A. Time curse knock down of SNF2PH in bloodstream forms. Rabbit anti-SNF2PH antiserum was used to validate protein depletion by western blot analysis.

B. SNF2PH depletion reduced growth of bloodstream form cells. Using inducible RNAi, three independent clones showed abrupt growth arrest upon 72h of doxycycline induction. Continuous line: Uninduced control / SNF2PH RNAi (Dox-). Discontinuous line: induced SNF2PH RNAi (Dox+).

C. Reduction of the active VSG221 expression is reported after 48h of RNAi induction in all clones analyzed, whereas inactive VSG expression is clone dependent. Triplicate fluorescent measures are represented as the average ±SEM from three independent SNF2PH RNAi clones for both Renilla (active VSG-ES promoter, upper panel) and Luciferase (inactive VSG-ES promoter, lower panel) reporters in a DRALI cell line.

D. SNF2PH KD reflects clone variability in inactive VSG transcription. Relative transcripts levels for FLuc reporter gene upstream an inactive VSG-ES assessed by RT-qPCR upon 48h of doxycycline induction. Data from three independent clones with standard error of the mean were normalized with U2 mRNA, transcribed by pol III. (Student's T-test: *$p<0.05$, **$p<0.01$,***$p<0.001$).

E. Scatter plot showing telomeric BES expression by RNA-seq analysis of SNF2PH depleted cells. Inactive VSG-ESs (BESs) upregulation occurs only in a subset of the inactive telomers, while other inactive BESs appear to be unaffected. Thus, BES deregulation seems to be variable within each of the clones analyzed, consistent with different reporter activities detected by the VSG-ES promoters (Fig S4C). SNF2PH is included as KD control. Data from two independent clones are presented compared with two RNAs isolated from the parental cell line.

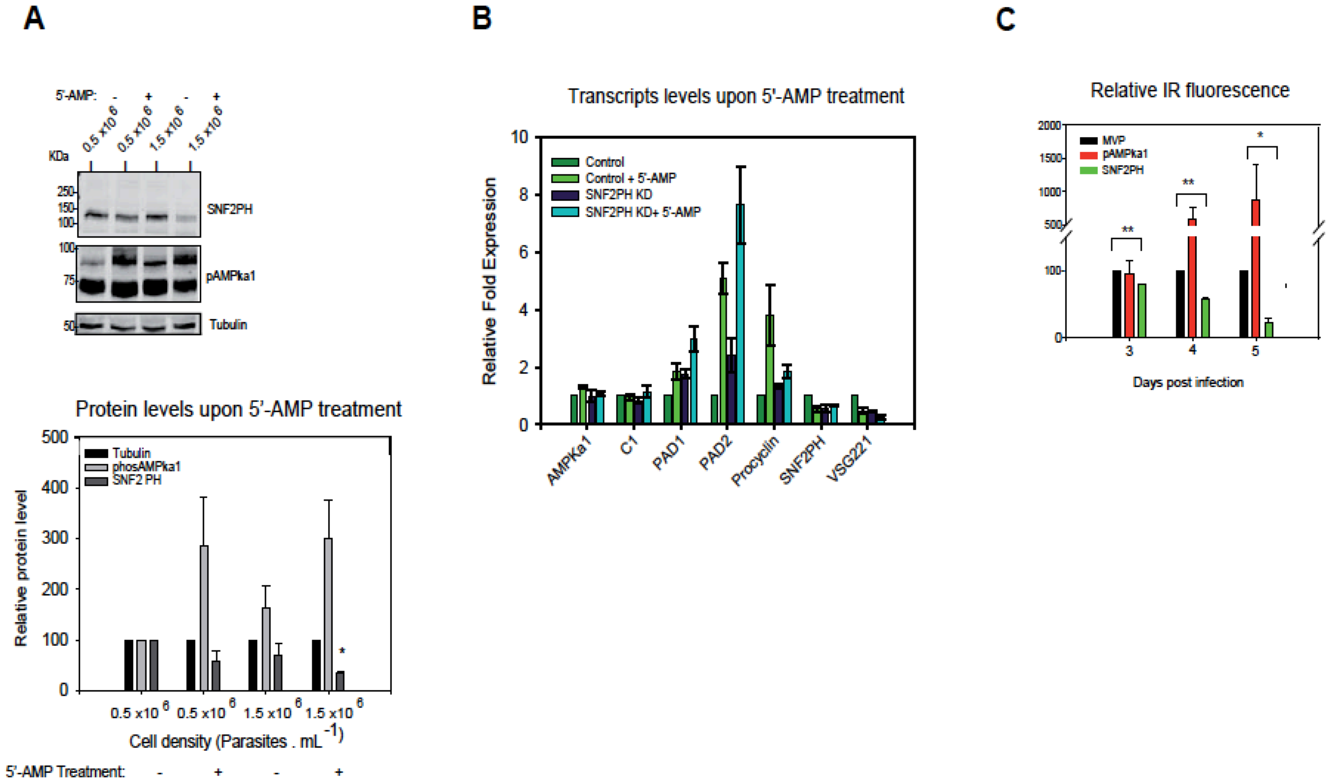

Appendix Figure S5. Downregulation of SNF2PH during stumpy form development.

A. (Upper panel) SNF2PH protein levels are reduced upon AMPK activation. Representative Western blots of cells treated with 1 μM AMP analogue for 18 hours compared with an untreated control at two different densities. (Lower panel) Histogram showing relative levels for phosphorylated AMPKα1 for the western blot shown in upper panel. (Student's T test): *p<0.001.

B. AMP analogue treatment positively regulates stumpy form gene expression of PAD genes in SNF2PH depleted cells. Relative transcript levels after treating control and SNF2PH depleted cells (24h) with or without 1μM AMP for 18 hours. Genes associated with stumpy form differentiation are represented as PAD1 and 2, procyclin and VSG221 and normalized with U2 as a housekeeping gene from three independent replicates.(Student's T-test): *p<0.05, **p<0.01.

C. SNF2PH is downregulated during *in vivo* stumpy form development in a pleomorphic cell line. Histogram showing the mean of SNF2PH expression and phosphorylated AMPKa1 in protein extracts of parasites isolated from 3-2 independent mice, the indicated day post infection (Dpi); the 3rd, 4th, and 5th. Western blot analysis was performed using the anti-SNF2PH antiserum and the phospho-AMPKα mAb anti-(Thr172) (40H9) (Cell Signalling) relative to a protein control (C1). Primary antibodies were incubated with tree pieces from the same blot corresponding to the approx.. sizes and developed using goat anti-rabbit IgG 800 Dylight (Thermo-Fisher). Membrane was scanned using an LI-COR Odyssey and analyzed using Odyssey IR imaging software 3.0.42. Error bars represent means ± SEM. Loading information: Dpi: days post infection. Ms: Mouse. (Student's T-test): *p<0.01, **p<0.001.
