# Supplementary material for: SUMOylated SNF2PH promotes variant surface glycoprotein expression in bloodstream trypanosomes
Source: EMBO Rep. 2019 Nov 6;20(12):e48029. doi: 10.15252/embr.201948029 (PMC6893287; doi:10.15252/embr.201948029)
Supplement: Supplementary file 2 — Expanded View Figures PDF [file EMBR-20-e48029-s002.pdf]

Expanded View Figures

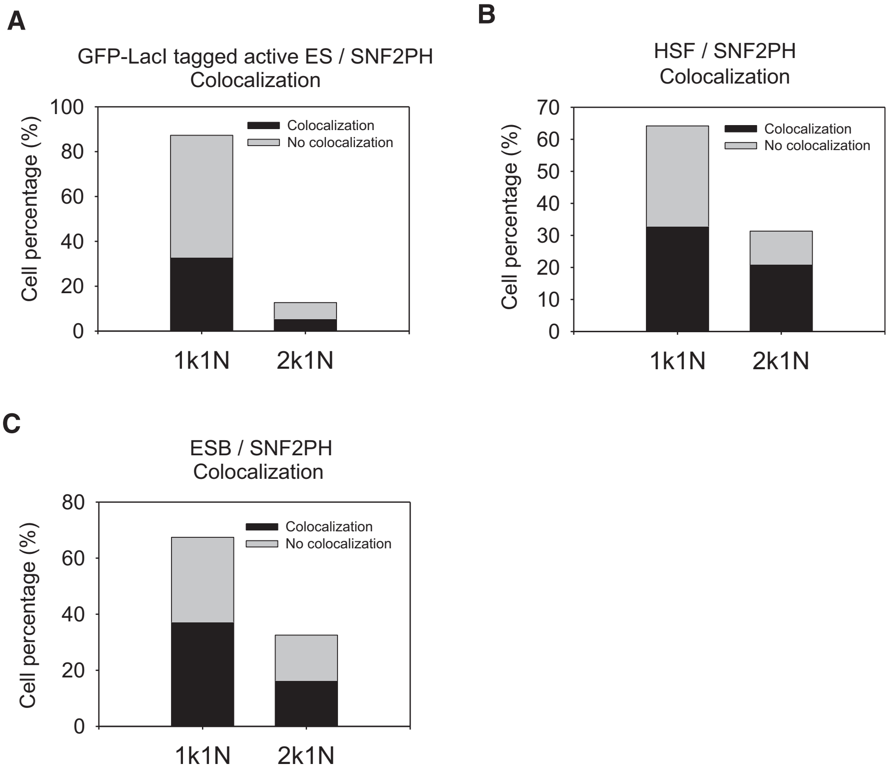

Figure EV1. SNF2PH statistical analysis of colocalization with the active VSG-ES, the ESB and the HSF.

A Histogram representing the proportion of cells that colocalize with the GFP-tagged active VSG-ES (BES1) the VSG221-ES was 38.17% (32.72% in 1K1N cells, 5.45% of 2K1N) (*n* = 55).

B Colocalization statistical analysis of the SNF2PH with respect to the HSF. Histogram of statistical analysis shows a colocalization of SNF2PH with the HSF in 32.83% of 1K1N cells and 20.9% of 2K1N cells (*n* = 67).

C Statistical analysis of ESB (YFP:TbRPB5z) and SNF2PH colocalization. Histogram of shows colocalization in 60.47% of analyzed cells (*n* = 43). In addition, percentage colocalization determined in each cell cycle phase is shown (37.21% (1K1N) + 16.27% (2K1N)).

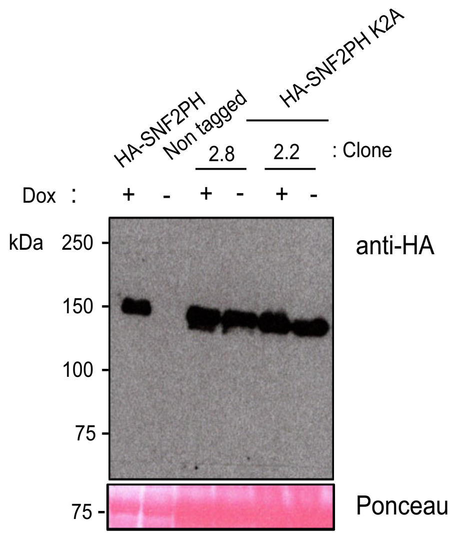

Figure EV2. Protein expression level of SNF2PH K2A is similar to the wild-type SNF2PH.

Cell lines stably transfected with a construct that allows doxycycline (Dox)-inducible expression of a 3HA-tagged protein (see Materials and Methods) were analyzed by Western blotting. Total protein extracts from cells expressing wild-type HA-SNF2PH and two independent clones expressing HA-SNF2PH with the K2A mutation, clones 2.2 and 2.8, were assessed using anti-HA antibodies mAb, Clone 3F10 (Roche). A cell line expressing HA-SNF2PH was utilized as control for HA-fusion protein detection. Untagged cell line is shown as negative control. Ponceau protein staining was used as loading control. Expression of HA-SNF2PH K2A was constitutive in 2.8 and 2.2 clones reason why both dox induced (+) and dox uninduced (—) extracts showed no differences in SNF2PH expression, suggesting that continued expression of mutant HA-SNF2PH K2A did not affected normal cell growth.

© 2019 The Authors

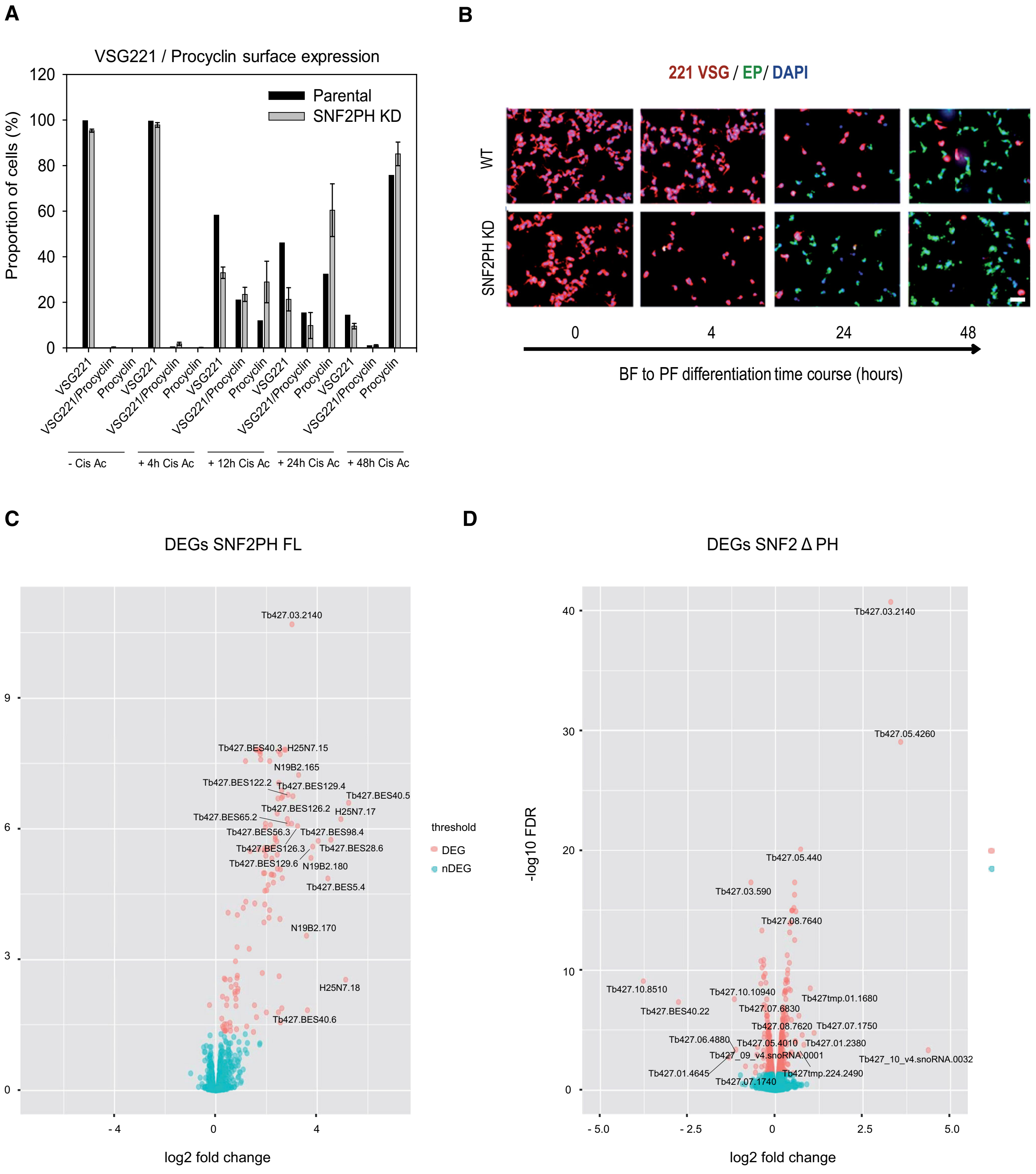

Figure EV3. SNF2PH is required for maintenance of bloodstream form stage-specific expression profile.

A Differentiation is accelerated when SNF2PH is depleted. Proportion of cells expressing VSG221 and procyclin at several time-points during differentiation. Error bars represent means ± SD of two representative biological replicates.

B Representative microscopy images at differentiation time-points. Double indirect 3D-IF was performed with rabbit anti-VSG221 (red), mouse anti-Procyclin (green) and DAPI staining (Blue) upon 3 mM of cis-Aconitate induction. Scale bar, 15 μm.

C Volcano plot showing upregulated genes (117) versus downregulated (1) in SNF2PH. The $\log_2$ FC and FDR ($-\log_{10}$ FDR) values are shown.

D Volcano plot showing most upregulated (397) and downregulated (340) genes in SNF2ΔPH considering FDR < 0.05.

 © 2019 The Authors
